# Does supplementing laying hen diets with a herb mixture mitigate the negative impacts of excessive inclusion of extruded flaxseed?

**DOI:** 10.5713/ab.22.0183

**Published:** 2022-11-14

**Authors:** Hossein Hosseini, Noah Esmaeili, Aref Sepehr, Mahyar Zare, Artur Rombenso, Raied Badierah, Elrashdy M. Redwan

**Affiliations:** 1 Department of Microbiology, Pathobiology and Basic Sciences, Faculty of Veterinary Medicine, Razi University, Kermanshah, 6714967346, Iran; 2 Institute for Marine and Antarctic Studies, University of Tasmania, Hobart, Tasmania, 7053, Australia; 3 Department of Land, Environment, Agriculture and Forestry, University of Padua, Padua, Italy; 4 South Bohemian Research Center of Aquaculture and Biodiversity of Hydrocenoses, Institute of Aquaculture and Protection of Waters, Faculty of Fisheries and Protection of Waters, University of South Bohemia, České Budějovice, 38925, Czech Republic; 5 CSIRO, Agriculture and Food, Livestock & Aquaculture Program, Bribie Island Research Centre, Bribie Island, Queensland, 4507, Australia; 6 Biological Science Department, Faculty of Science, King Abdulaziz University, Jeddah 21589, Saudi Arabia; 7 Medical Laboratory, King Abdulaziz University Hospital, King Abdulaziz University, Jeddah 21589, Saudi Arabia; 8 Protein Research Department, Genetic Engineering and Biotechnology Research Institute, City of Scientific Research and Technological Applications (SRTA-City), New Borg EL-Arab, Alexan-dria 21934, Egypt

**Keywords:** Cholesterol Level, Egg Enrichment, Flaxseed, Herbal Medicine, Lipid-lowering Effect

## Abstract

**Objective:**

This study investigated the effects of extruded flaxseed with and without herbs mixture on egg performance, yolk fatty acids (FAs), lipid components, blood biochemistry, serological enzymes, antioxidants, and immune system of Hy-Line W-36 hens for nine weeks.

**Methods:**

Two hundred forty laying hens were randomly distributed to eight treatments, resulting in six replicates with five hens. Graded levels of dietary extruded flaxseed (0, 90, 180, and 270 g/kg) with and without herbs mixture (24 g/kg: garlic, ginger, green tea, and turmeric 6 g/kg each) were designed as treatments.

**Results:**

The two-way analysis of variance indicated that hens fed herbs mixture had a higher value of egg production, yolk high-density lipoprotein (HDL), superoxide dismutase, glutathione peroxidase, and white blood cell and lower contents of yolk cholesterol, glucose, and blood low-density lipoprotein than those fed diets without herb mixtures (p<0.05). The Flx27 (270 g/kg flaxseed) (153.5 g/kg n-3 FAs) and Flx27+H (270 g/kg flaxseed plus 24 g/kg herbs mixture) (150.5 g/kg n-3 FAs) groups were the most promising treatments in terms of yolk n-3 FAs content. In-teraction effect (herbs-flaxseed) for blood cholesterol, HDL, malondialdehyde, glutaredoxin, alanine transaminase, (ALT), aspartate transaminase (AST), haemoglobin and immune parameters was significant (p<0.05). The results showed layers fed herbs mixture (Flx9+H, Flx18+H, and Flx27+H) had a better value of total antibody, immunoglobulin M, immunoglobulin G, ALT, AST, and blood HDL as compared with representative flaxseed levels without herbs.

**Conclusion:**

High inclusion levels of extruded flaxseed (270 g/kg) without herbs to enrich eggs with n-3 appears to impair the antioxidant system, immunohematological parameters, and sero-logical enzymes. Interestingly, the herbs mixture supplementation corrected those effects. Therefore, feeding layers with flaxseed-rich diets (270 g/kg) and herbs mixture can be a promising strategy to enrich eggs with n-3 FAs.

## INTRODUCTION

Fatty acids (FAs) are major components of cell membranes and act as gene regulators of many metabolic processes. Among them, n-3 FAs play key roles in the immune system, receptor expression, blood coagulation, vascular resistance, enzyme activity, cell proliferation, and differentiation [1]. Therefore, medical practitioners and researchers strongly suggest consuming foods rich in n-3 FAs to contribute to a healthy lifestyle. Western diets contain a high level of n-6 FAs, and the ratio of n-6 to n-3 polyunsaturated fatty acids (PUFAs) has risen from 1:1 in the last century to 10:1 in recent years [1]. Consumption of fast foods and diets including high n-6 fats and few contents of aquaculture products in food baskets (they are a rich source of n-3 FAs) caused this problem. This shift in FA content corresponds with a large rise in the prevalence of overweight and obesity. One of the sustainable and easiest ways to alleviate this problem is to enrich eggs (one of the most important foods for humans) with n-3 FAs [2]. The egg production in 2019 passed 80 million tonnes. Therefore, there is a great opportunity to improve the quality of human foods via egg enrichment. Although marine ingredients (for example, fish oil) can be the best option to provide n-3 FAs, their availability and limited resources in the marine environment are big obstacles. This limitation has caused researchers and farmers to test cheaper and more sustainable ingredients that contain high contents of n-3 FAs, like plants and seeds. Flaxseed (*Linum usitatissimum*) is the most common ingredient to enrich eggs with n-3 FAs [3]. This ingredient has been the cheapest and is available with a consistent quality source of n-3 FAs. Antinutritional factors found in flaxseed meals include tannin and cyanogenic glycosides (linustatin, neolinustatin, and linamarin) [4], which can negatively affect animals when added at high levels to diets.

Herbal medicines contain various compounds, including phenols, tannins, alkaloids, terpenoids, and polysaccharides [5]. Thousands of herb species in nature can potentially result in positive impacts on animal growth and health. However, these herb species have been tested in animal species, and there is an endless opportunity to discover new features of thousands of herbs. These herbs can promote the performance of hens and improve antioxidant capacity and flesh quality; and stimulate digestive enzymes, appetite, and the immune system [6]. In our previous studies, we observed the positive effects of garlic (*Allium sativum*) [7,8], barberry (*Berberis vulgaris*) [9,10], figwort (*Scrophularia striata*) [11] and dill (*Anethum graveolens*) [12] on animal growth and health. In chicken, among a long list of herbs, garlic, ginger (*Zingiber officinale*), turmeric (*Curcuma longa*), and green tea (*Camellia sinensis*) have been widely studied [6,13–16]. These herbs also are famous for their lipid-lowering effect on different animals, including poultry [17–19].

In our previous study on hens, different processing methods and levels of flaxseed was tested, and hens fed diets containing 90 g/kg extruded flaxseed had higher performance regarding egg performance, lipid components, FAs concentrations in yolk and blood, and antioxidant system compared to the whole and milled flaxseed-based diets [20]. In the present follow-up research, we aimed to i) test higher levels of extruded flaxseed up to 270 g/kg, focusing on the health status and n-3 FAs eggs enrichment of hens, and ii) test the effects of herbs mixture (garlic, ginger, turmeric, and green tea) supplementation in haematology, immune response, and antioxidant system of layers. Despite the fact that earlier investigations had looked into the impacts of flaxseed in different levels and forms on performance and egg enrichment with n-3 FAs in hens, there is no study regarding the beneficial effects of herbs on this process. Further, no study comprehensively investigated different physiological and health parameters of hens under feeding with excessive levels of flaxseed. Therefore, for the first time, we investigated the effects of graded levels of extruded flaxseed with and without the herbs mixture on laying performance, FAs profile, egg quality, blood parameters, lipid metabolism, antioxidant system, serological enzymes, haematology, and immune system of hens.

## MATERIALS AND METHODS

### Birds, farming, and experimental design

The experiment took place at a large commercial egg-producing farm (Morghineh Markazi Farm, Arak, Iran) [21]. For two weeks, all hens were fed the control feed (Table 1) to let them adjust to the experimental conditions, and then, 240 Hy-Line W-36 hens (50 weeks old) were assigned to the experimental pens at random. Plus control diet, three graded levels of extruded flaxseed (brown variety) with and without 24 g/kg herbs mixture (H) (24 g/kg, containing garlic, ginger, turmeric, and green tea powder; 6 g/kg from each) were formulated (Table 1). The eight treatments were control, Flx9 (90 g/kg flaxseed), Flx18 (180 g/kg flaxseed), Flx27 (270 g/kg flaxseed), control+H (24 g/kg H), Flx9+H (90 g/kg flaxseed +24 g/kg H), Flx18+H (180 g/kg flaxseed+24 g/kg H), and Flx27+H (270 g/kg flaxseed+ 24 g/kg H). In this study, the extrusion process of flaxseed was carried out following a single screw extruder (Yemmak Extruder, Bandirma, Turkey), as detailed in our previous works [20,21]. The chickens were kept in 48 steel pens (each with five hens) with concrete flooring, and the photoperiod was adjusted in a 16:8 (light:dark) schedule. During the experiment, the room temperature was 20°C±2°C, and the relative humidity was approximately 40%±10%. All diets were isonitrogenous and isoenergetic, and water and feed were freely available (160 g/kg protein and 13 MJ/kg smetabolisable energy) (Table 1). An Institutional Animal Care and Use Committee of Tarbiat Modares University (281-1385) approved all experimental protocols [21,22].

### Chemical composition and bioactive compounds measurements

The chemical composition of diets was analysed using AOAC methods (AOAC 2000), as previously reported in detail [23]. Briefly, crude protein was determined by the Kjeldahl method, using an automatic Kjeldahl system (Kjeltec Analyser unit 2300; Hillerod, Sweden). Crude lipid was analysed with the Soxhlet extraction method (Soxtec 2050 FOSS, Effretikon, Switzerland). Moisture was determined by drying samples in an oven at 105°C for 12 h and a Nabertherm muffle furnace (Nabertherm GmbH, Lilienthal, Germany) was used to determine ash (550°C for 4 h). Nitrogen-free extract plus fibre, representing carbohydrate, was calculated using the formula: carbohydrate = 100–(protein+lipid+ash+moisture). The gross energy of the diet was calculated according to the National Research Council [24]:


Energy (MJ/kg)=(protein×23.6 kJ/g)+(fat×39.5 kJ/g)+(carbohydrate×17.2 kJ/g)

For measuring curcumin, experimental diets were analysed by high-performance liquid chromatography (HPLC) using a UV detector. Pure curcumin was dissolved in methanol and then injected into HPLC for making a standard curve [25]. An HPLC method using butyl parahydroxybenzoate as the internal standard was used for the quantification of allicin. The technique with full details was reported elsewhere [26]. Total phenolics, total tannin, and total flavonoids were determined by Folin–Ciocalteu method and the aluminium chloride method, respectively, which were explained in detail elsewhere [27].

### Sampling and measurements

Production performance of hens, such as egg production percentage, egg weight, feed intake, and feed conversion ratio, were measured weekly from daily collected eggs. Also, 24 eggs (no shell defects, cracks, or double-yolk eggs) were randomly collected from each treatment in the last two weeks (one per day) for egg quality evaluation. An Egg Multi Tester (Egg Multi Tester EMT-5200; Robotmation, Tokyo, Japan) was used to measure egg weight, Haugh unit [28] and height albumin. Shell thickness was measured at three different locations (middle, broad, and narrow ends) using a digital micrometre gauge (0–1″ Digital Electronic Micrometer; iGaging, Los Angeles, CA, USA), and mean values were considered as thickness. The egg yolk was extracted from the egg carefully by a yolk separator, weighed, and stored at −20°C±2°C for further analysis. Finally, the eggshell breaking strength was measured with an eggshell force gauge (Model-II; Robotmation, Tokyo, Japan).

### Blood collection

At the end of the experiment, two millilitres of blood were taken from each layer using sterilised syringes and needles, blood was drawn from the brachial wing vein (sterile 3 mL disposable syringes with needle size 21 gauge and 3.8 cm). Serum was separated by centrifugation at 1,150 g for 10 minutes after one hour of standing at room temperature and clotting. Serum samples were stored at −80°C until further analysis. Six samples from each treatment were taken on sampling day, and two of them were randomly pooled into one tube to make four replicates for each treatment (1+1, 1+1, 1, 1).

### Measurements of lipid components in egg and blood biochemistry parameters

According to Elkin and Rogler [29], 1 g of egg yolk was homogenised with 15 mL of chloroform-methanol 2:1 (by volume), sonicated, and filtered to extract the lipid. Using commercially available reagent kits, total cholesterol (CHO), high-density lipoprotein (HDL), triglyceride (TG), and low-density lipoprotein (LDL) levels were determined enzymatically (Parsazmon Company; Tehran, Iran).

Total protein, albumin, globulin, glucose, CHO, TG, HDL, and LDL were analysed with commercial kits (Pars Azmun, Iran). The producer of measurement of any parameter was done according to the available protocol in the kit pack.

### Fatty acids analyses in yolk egg

The FAs profile was determined via extraction of lipids from the egg yolk and diets with a chloroform and methanol mixture (2:1 volume) and are shown in Table 2. We used gas chromatography (GC) (CP 3800; Varian Analytical Instrument; Walnut Creek, CA, USA) equipped with a flame ionisation detector fitted with a permanently bonded polyethylene glycol, fused silica capillary column (PBX70; 120 m ×0.25 mm internal diameter, film thickness 0.25 μm; SGE Analytical Science; Melbourne, Australia) to separate the resultant fatty acid methyl esters (FAMEs). The complete procedure was detailed in our previous studies [30,31].

### Antioxidant activity and liver enzymes

Serological enzymes, including lactate dehydrogenase (LDH), alkaline phosphatase (ALP), aspartate transaminase (AST), and alanine transaminase (ALT), were determined in plasma samples by auto analyser spectrophotometer instrument (Costa Brava 30, 08030; BioSystems S.A., Barcelona, Spain). The activity of antioxidant enzymes, including malondialdehyde (MDA), superoxide dismutase (SOD), glutathione peroxidase (GPx), glutathione reductase (GRx), and catalase (CAT), were measured according to methods that were mentioned with details elsewhere [20].

### Immune response and haematological parameters

For measuring immunoglobulin G (IgG), and IgM, a total antibody titer against the sheep red blood cell (SRBC) was used as described in detail earlier [32]. To determine components of the response to SRBC (IgG and IgM), the IgM antibody, which is sensitive to mercaptoethanol, and could therefore be calculated by isolating the resistant antibody to mercaptoethanol (IgG) (total response – IgG = IgM) was used.

The red blood cell (RBC) and white blood cell (WBC) were determined by a hemocytometer method using a Natt-Herrick solution; hematocrit values and haemoglobin amounts were measured by microhematocrit and a commercial kit, respectively (Pars Azmun, Iran). Blood indices were calculated according to the below formulas:


$$\begin{array}{l} \text{Mean corpuscular volume }(\text{MCV})\;(\text{fL})=[\text{Haematocrit}/(\text{RBC};\;10^{6}/{\text{mm}}^{3})]\times 10\\ \text{Mean corpuscular haemoglobin }(\text{MCH})\;(\text{pg})=[\text{Haemoglobin}/(\text{RBC};\;10^{6}/{\text{mm}}^{3})]\times 10\\ \text{MCHC }(\%)=[\text{Haemoglobin}/\text{Haematocrit})]\times 100\\ \text{Blood performance }(\text{BP})=\text{Ln haemoglobin }(\text{g}/\text{dL})+\text{Ln haematocrit }(\%)\\ +\text{Ln RBC}(10^{5}/{\text{mm}}^{3})+\text{Ln WBC }(10^{3}/{\text{mm}}^{3})\\ +\text{Ln total protein }(\text{g}/\text{L}) \end{array}$$[
33]

### Statistical analyses

The study was performed under a completely randomised design. Data were analysed by two-way analysis of variance (ANOVA) after checking the normality and homogeneity of variance using SPSS software (release 22.0 for Windows). For two-way ANOVA, the Herbs effect (with vs without herbs mixture) and the Flaxseed effect (different flaxseed levels) were considered as the main factors. When the p-value of interaction was significant (p<0.05), we compared treatments in original (non-pooled) data in different levels of flaxseed and with and without herbs mixture via Tukey and independent sample t-test, respectively. When the p-value of interaction was insignificant, treatments in pooled data via independent sample t-test and Tukey were compared (Table 3).

## RESULTS AND DISCUSSION

### Egg performance

Although the first goal of adding flaxseed to diets is to enrich eggs with n-3 FAs, egg performance can be simultaneously improved. The addition of herbs can further improve egg performance. Egg performance is one of the most important phenotypes in hen production. Given the millions tone of produced eggs each year, any few percentage increases in these parameters can end up with millions of dollars in benefits for farmers. In the current research, shell thickness, shell strength, egg weight, Haugh unit, yolk weight, feed intake, egg mass, and feed conversion ratio were not significantly different among treatments (Table 4). Egg production was significantly affected by herb supplementation resulting in higher values (77.5%) than those without herbs (73.97) (p< 0.05) (Table 3). Egg production is an important economic factor, and the inclusion of up to 270 g/kg of extruded flaxseed plus herbs mixture performed equally to the control. It is worth highlighting that pre-processing flaxseed via extrusion helped to reach a high inclusion level. In our previous study with similar experimental conditions and the same line of hens (Hy-Line W-36), an adverse effect of whole seed or milled flaxseed on egg performance of layers was observed [21]. Deactivation of antinutritional factors and releasing of intercellular oil from flaxseed under high pressure and temperature conditions during extrusion processing increase bioavailability and utilisation by animals [34]. These benefits, along with the positive effect of herbal medicine, enabled the inclusion of higher dietary levels of extruded flaxseed (up to 270 g/kg) without any adverse effect on egg performance. Layers in the Flx27+H treatment probably spared energy for maintenance, tackling nutritional stress and other non-productive pathways, and thus, channelling it for egg production. However, without the herbs mixture, the inclusion of extruded flaxseed was limited to 180 g/kg. Similarly, other studies observed no adverse or positive effects of dietary supplementation of extruded flaxseed up to 200 g/kg on egg production [35]. On the other hand, whole flaxseed can only be included in hens' diets up to 100 g/kg without impairment [36]. Antinutritional factors in whole flaxseed appear to affect digestion and absorption of nutrients and, eventually, decrease egg performance. Furthermore, n-3 FAs, which are plentiful in flaxseed, are necessary for optimal growth and development, high production rate, and reproductive performance [37]. Any ingredients containing a high level of n-3 FAs can improve egg production as long as they do not impair layer growth and health. Similar to the presented data, the positive effect of adding herbs on egg production was reported. Although these results show that the selected herbs could positively impact egg performance, more studies at classic and molecular levels are required to illustrate which potential pathways and biological mechanisms are responsible for these positive responses.

### Yolk fatty acids profile

As most FAs are deposited in yolk, the profile of FAs can reflect the lipid nutritional compositions of eggs. Therefore, in the current study, we measured FAs profile in the yolk to see how the egg enrichment with flaxseed worked. FAs profile of yolk reflected the dietary treatments, with hens fed flaxseed-based diets displaying higher levels of n-3 FAs compared to those provided in the control (Table 5), which is consistent with the literature [35,38]. Flaxseed effect for most fatty acids such as saturated fatty acids (SFAs), monounsaturated fatty acids (MUFAs), C18:2n-6, C18:3n-3, C20:4n-6, C20:5n-3, C22:6n-3, PUFA, n-3, and n-6 was significant (p<0.05). Flx27+H and Flx27 were the treatments with the most n-3 FAs content in the yolk. This result is in line with other findings [39]. As observed, the herbs mixture did not affect yolk FA composition. Researchers recommend a balanced dietary n-6 to n-3 ratio as a human health promoter [1]. A ratio of less than 4:1 has been proposed as a standard number to ensure cardiovascular system performance, anti-inflammatory benefits, and atherosclerosis reduction [1].

### Lipoproteins, CHO, and TG in the yolk

Egg yolk lipids and lipoproteins are responsible for cell membrane formation and provide the primary energy source for cerebral development [40]. In addition to n-3 FAs content in the yolk, lipid composition is paramount as it directly affects metabolism. According to Tables 3 and 6, Herb's effect on yolk CHO, total CHO, and yolk HDL were significant, and layers fed herb-supplemented diets had a lower value of yolk CHO (10.62 vs 12.37 mg/g yolk), total CHO (210.4 vs 239.1 mg/g yolk), and higher contents of yolk HDL (4.40 vs 3.36 mg/g yolk). Further, hens fed FLX18s (11.01 mg/g yolk) and FLX27s (10.63 mg/g yolk) diets had lower CHO in the yolk than the controls groups (p<0.05). Results show that both flaxseed and herbs mixture have a role in decreasing CHO contents. Literature reports inconsistent results such as no change [41], decrease [36,38], and increase in yolk HDL [42] when layers are fed flaxseed diets in different forms. Various levels of flaxseed, processing types, nutritional quality (e.g. lipid composition) and experimental design can be possible reasons for the observed inconsistency. Several investigations found the CHO-lowering effect of herbs on egg yolks. For example, dietary supplementation with herbs [43,17] resulted in this effect in hens. The positive impacts of flaxseed and herbs can be due to the inhibitory effect of phenolic compounds on 3-hydroxy-3-methylglutaryl coenzyme A, which is one of the most important components of CHO synthesis [44]. Further, HDL metabolism, which is involved in reverse CHO transport, can regulate yolk CHO contents [44]. In summary, Flx18+H and Flx27+H groups resulted in better yolk lipid composition than the rest of the treatments. However, more investigations are necessary to get insights into the underlying mechanisms modifying yolk lipid content by dietary herbs and flaxseed and their interaction.

### Blood biochemistry parameters

Blood parameters are a good indicator of the welfare of laying hens as they indicate health status and metabolism hemostasis [45]. According to Tables 3 and 6, there were no significant differences among treatments in total protein, albumin, globulin, albumin/globulin ratio, TG, and LDL. The two-way ANOVA showed hens fed dietary herbs had lower values of glucose (258.7 vs 283.7 mg/dL) and blood LDL (66.2 vs 79.5 mg/dL) than those fed diets without herbs (Table 3). Further, blood LDL levels in flaxseed groups were lower than controls showing a positive effect of flaxseed on CHO metabolism. The interaction effect for blood CHO and HDL was significant (Table 6). While there was no significant difference in +H treatments, Flx27 had lower and higher contents of CHO and HDL, respectively, than the control. Further, blood HDL in all four levels of flaxseed in +H treatments were higher than those fed diets without herbs mixture (p<0.05). These results confirm the hypothesis about the CHO-lowering effect of these four herbs. In our previous studies, we observed that the extrusion process improved total proteins and decreased CHO content in the blood [20]. Regarding the current study, extruded flaxseeds did not negatively affect blood biochemistry and positively affected HDL and CHO levels. Collectively, these findings might indicate the important role of the quality of flaxseed in terms of processing rather than the levels in blood biochemistry. More studies are required to test this hypothesis, whether blood biochemistry has a relationship with the level and/or quality of ingredients.

Herbs are well-known to promote total proteins and albumin to globulin ratio in poultry, which was reviewed elsewhere [46]. However, such positive effects were not observed in the present study. It can be due to differences in the age of poultry, herb species, other ingredients in diets, health status, etc. Other studies observed similar results when feeding layers with supplemented diets with herbs [18,47] improved CHO and HDL levels. The CHO-lowering effect in poultry driven by dietary herbs and seeds appears to be associated with two mechanisms. Firstly, crude fibre can decrease CHO absorption and faecal sterol excretion [48]. Secondly, CHO is known to induce cholic and deoxycholic bile acid production by hepatocytes. These acids pass through the small intestine and are absorbed before being sent to the liver. Eventually, as CHO is used for bile acid synthesis, an increase in bile acid production decrease serum CHO concentration [49]. Even after many years of research, the exact mechanism behind the lipid-lowering effect of herbs is yet to be determined [4]. More research at the molecular level is required to discover potential genes and proteins as biomarkers of the lipid-lowering effect. In the context of blood biochemistry, the Flx9+H and Flx27+H groups displayed more promising results than the rest of the dietary treatments.

### Antioxidant and serological enzymes in the blood

According to Table 3, hens fed herbs diets had higher values of SOD (93.67 vs 71.46 U/mL) and GPx (1,021 vs 885 U/mL) than individuals fed diets without herbs. The interaction effect of two-way ANOVA was significant for MDA and GRx parameters. Supplementing extruded flaxseed improved MDA in +H treatments, and they had a lower value than the control+H group (Table 7). Regarding GRx, there was no significant difference in +H treatments and further, layer fed dietary Flx27 had a lower value than control, Flx9, and Flx18 (p<0.05). As observed, both extruded flaxseed levels and herbs had significant impacts on the antioxidant system of hens. Extruded flaxseed diets, especially Flx27s, had a high content of n-3 FAs (311.8) and, eventually, yolk (153.5). These fatty acids are susceptible to lipid oxidation more than n-6 FAs [50]. This issue might increase the susceptibility of hens to lipid oxidation. On the other hand, the antioxidant effect of flaxseed was demonstrated in several studies [51]. Enrichment of eggs with flaxseed should be thoughtfully considered as excessive levels induce oxidative stress, and lower inclusion levels fail to result in a positive impact on egg enrichments. However, this effect was corrected by supplementing the herbs mixture, which likely protected hens from oxidative stress generated by the high inclusion of extruded flaxseed (270 g/kg). The antioxidant activity of herbs has been well-known since the 1970s [52]. Although these four herbs (garlic, ginger, green tea, and turmeric) were chosen because of their lipid-lowering effects, they have also been introduced as an antioxidant agent in poultry studies [53]. Additionally, our previous research concluded that the extrusion method decreased lipid peroxidation in hens [20]. In conclusion, supplements such as herbs that can improve the antioxidant system are necessary for enriching eggs with n-3 FAs.

Serological enzyme concentrations in serum or plasma, such as LDH, ALP, AST, and ALT, are frequently used to assess liver function in chickens [54]. They have been reliable biomarkers of the health and nutritional status of animals. If the ALT, AST, and LDH elevated and ALP declined under any experimental conditions, it can be hypothesised that the health status of the animal has not been at an optimum condition. According to Table 7, there was no significant difference in the ALP and LDH contents among layers fed various diets in this trial. The interaction effect was significant for AST and ALT, and those fed flaxseeds and without herbs diets (Flx9, Flx18, and Flx27) had a higher value than the control. However, such effects were not observed in +H groups showing that herbs positively affected these two parameters.

The results were compatible with antioxidant enzymes showing that hens fed extruded flaxseed diets without herbs were likely affected by oxidative stress. These results are consistent with another study on hens [55]. Flaxseeds contain hydrogen cyanide, which is a toxin that accumulates in the liver. The liver then tries to detoxify them and eventually causes an increase of serological enzymes in the blood [56]. Dietary hydrogen cyanide was not analysed, but it is possible that this phenomenon happened in the present study. Another interesting result was the lowering effect of herbs on serological enzymes. The protective effects of garlic, ginger, green tea, and turmeric against the elevation of serological enzymes were reported in poultry [57]. Accordingly, the Flx9+H, Flx18+H, and Flx27+H groups had better performance regarding antioxidant activities and liver enzymes.

### Hematology and immune response in the blood

Haematology parameters are important for monitoring health status during environmental and nutritional stresses, and they are closely related to the immune response. According to Table 3, the herbs effect was significant for WBC, and those fed herbs diets had a higher value than hens who ate dietary without herbs (p<0.05). All measured immune response parameters were remarkably affected by both dietary extruded flaxseed and herbal medicines supplementation, as the interaction effect was significant for them. Generally, hens fed +H diets had higher values of these parameters (total antibody, IgG, and IgM after 24 and 42 days) than the without herbs groups in each level of flaxseed. The best treatment in this regard was Flx27+H displaying higher values of total antibody, IgG, and IgM after 24 and 42 days than Flx27 (p< 0.05). While there was no difference between Flx27+H and control+H treatments in the abovementioned parameters, the without herbs group experienced changes. The Flx27 in all immune parameters had lower values than control, showing that 270 g/kg flaxseed was excessive and an immune suppressor but not when herbs mixture was added. Blood performance was introduced as a new haematological parameter, which is calculated from the RBC, WBC, haematocrit, haemoglobin, and total protein [33]. We calculated this parameter, and interestingly shows the health status of hens, and the Flx27 group had the lowest value. In the present study, a high level of extruded flaxseed (270 g/kg) negatively affected immunohematology parameters and antibody response (Table 8). However, the herbs mixture with immunostimulatory effects prevented immunity suppression in the Flx27+H group. Adding flaxseed up to 50 g/kg in chickens [58] had a positive effect on the immune system and antibody titer. However, to the best of our knowledge, no study investigated the impact of such a high level of flaxseed on hens' immune system, and this study is the first report. Also, we have not found any investigation related to the effect of flaxseed on the haematology of layer hens. Improvement of haematology parameters by feeding herbs and their immunostimulatory effects have been reported. For example, feeding hens with herbs [13,27] resulted in positive impacts such as increased WBC, haemoglobin and hematocrit. As suggested in the last section, considering the antioxidant system and immune response are necessary for the context of egg enrichment with n-3 FAs. Suppressing the immune system at the expense of increasing n-3 FAs content in eggs can affect the economic and productivity gains and impair the welfare and health status of hens. To sum up, adding 270 g/kg of extruded flaxseed plus herbs mixture did not suppress the immune system of hens.

In conclusion, n-3 FAs enrichment of eggs by high dietary content (270 g/kg) of extruded flaxseed with (Flx27+H group) and without (Flx27 group) the herbs mixture was successful. Layers could consume dietary Flx27+H without any adverse impacts on lipid components in yolk and blood, antioxidant enzyme activities, liver enzymes, immune system and haematological parameters but not the Flx27 group. For the first time in poultry nutrition, we demonstrated adding high dietary levels of flaxseed without impairing layer performance and health status thanks to the supplementation of herbs. The herbs potentially have several benefits for farm animals to improve growth and health status. Therefore, we suggest the suitability of the Flx27+H diet to use in commercial laying hens farms. However, more investigations are required to test the performance of this diet on different farms and experimental conditions for layers of different ages. Additionally, we recommend more attention to the antioxidant activity, liver enzymes and immune system of layers fed diets rich in flaxseed. Experiments to examine the molecular processes behind the beneficial benefits of herbs mixture on poultry metabolism and health are also advised.

## Figures and Tables

**Table 1:** Dietary formulation and chemical composition of diets containing graded extruded flaxseed levels and herbals mixture

Items	Control	Flx9	Flx18	Flx27	Control+H	Flx9+H	Flx18+H	Flx27+H
Ingredients	------------------------------------------------------------------ g/kg, as fed basis --------------------------------------------------------------							
Corn meal	520	500	482	422	520	500	482	422
Soybean meal	241	201	141	111	241	201	141	111
Extruded flaxseed meal	0	90	180	270	0	90	180	270
Soybean oil	42	12	0	0	42	12	0	0
Other ingredients^1)^	127.1	127.1	127.1	127.1	127.1	127.1	127.1	127.1
Herbs mixture (H)	0	0	0	0	24	24	24	24
Filler (starch)	24	24	24	24	0	0	0	0
Chemical composition								
Crude protein (g/kg)	156.9	158.7	160.3	161.0	156.4	160.5	156.2	154.7
Total lipid (g/kg)	72.2	70.6	81.3	94.0	71.9	71.5	79.8	92.5
Carbohydrate (g/kg)	539.9	546.3	531.6	510.5	544.3	538.6	543.9	520.1
Ash (g/kg)	134.6	130.9	130.0	134.2	132.0	129.9	130.5	133.6
Dry matter (g/kg)	903.6	906.5	903.2	899.7	904.5	900.5	910.4	900.9
Metabolizable energy (MJ/kg)^2)^	12.99	13.05	13.23	13.36	13.03	13.02	13.27	13.32
Allicin (mg/kg)	1.5	1.9	2.1	1.8	489.4	487.5	494.3	500.2
Total phenolics (mg/kg)	2,231	2,300	2,287	2,259	2,319	2,364	2,352	2,371
Total flavonoids (mg/kg)	895	996	977	965	1,348	1,350	1,346	1,295
Total tannin (mg/kg)	2,201	2,049	2,028	2,021	2,782	2,643	2,577	2,599
Curcumin (mg/kg)	10.5	11.2	10.3	8.7	169.4	167.4	164.9	165.2

1) Other ingredients include Wheat bran 45.9 g, dicalcium phosphate 15 g, calcium carbonate 100 g, NaCl 3.8 g, DL-methionine 1.3 g, vitamin supplement 2.5 g, and mineral supplement 2.5 g, lysine 2 g. Minerals provided per 1 kg of diet: Fe (50 mg), Zn (50 mg), Se (0.2 mg), Co (0.2 mg), Cu (10 mg), Mn (100 mg), I (1 mg), choline chloride (250 mg) vitamins provided per 1 kg of diet: vitamins A, 10,000 IU; D_3_, 5,000 IU; E 50 IU; K_3_, 2 mg; B_1_, 2 mg; B_2_, 4 mg; B_3_, 30 mg; B_5_, 20 mg; B_6_, 2 mg; B_9_, 2 mg; B_12_, 0.01 mg; C, 1 mg; 100 g; inositol, 10 mg. Herbs mixture (24 g/kg) containing garlic, ginger, turmeric, and green tea powder; 6 g/kg from each (6×4 = 24).

2) First gross energy was calculated according to coefficients for crude protein, lipid and carbohydrate of 23.6, 39.5, and 17.2 MJ/Kg, respectively [25]. Then, constant factor of 0.82, which was suggested by the NRC was used for calculation of smetabolisable energy [25].

Dietary treatments with different levels of extruded flaxseed with and without herbal medicine: Control, Flx9 (90 g/kg flaxseed), Flx18 (180 g/kg flaxseed), Flx27 (270 g/kg flaxseed), plus these four treatments that included 24 g/kg herbs mixture as well.

Chemical compositions of ingredients: corn meal contained 100.3 g/kg protein, 45.3 g/kg lipid, 15.4 g/kg ash; soybean meal contained 420.8 g/kg protein, 17.5 g/kg lipid, 65.5 g/kg ash; wheat bran contained 145.6 g/kg protein, 42.1 g/kg lipid, 55.0 g/kg ash; and flaxseed meal contained 243.4 g/kg protein, 351.2 g/kg lipid, 41.8 g/kg ash.

**Table 2:** Fatty acid compositions of test diets with different levels of flaxseed and without herbal medicines fed to laying hen for nine weeks

Fatty acids	g/kg fatty acid methyl esters (FAMEs)								SDM

	Control	Flx9	Flx18	Flx27	Control+H	Flx9+H	Flx18+H	Flx27+H	
C14:0	8.6	17.4	18.6	19.7	8.9	17.9	15.4	20.0	0.6
C16:0	128.6	75.4	72.1	66.5	127.4	74.3	79.4	63.1	15.4
C18:0	58.4	38.5	36.9	33.0	60.3	33.6	30.2	30.7	9.3
SFAs^1)^	175.5	156.3	140.4	135.1	176.5	160.2	138.4	131.6	21.5
C16:1n-7	3.6	15.9	20.8	26.3	3.3	13.6	17.9	24.5	5.4
C18:1n-9	273.1	296.4	300.7	321.4	268.4	289.1	305.4	320.8	29.0
C20:1n-1	7.0	6.1	5.8	6.2	7.3	6.5	5.1	5.2	0.4
C22:1n-9	9.7	8.2	7.5	7.5	9.5	8.3	6.9	6.8	0.4
MUFAs^2)^	289.3	319.6	321.5	335.0	280.5	323.5	320.4	334.1	28.7
C18:2n-6	376.7	205.5	204.0	201.8	371.2	210.4	209.3	200.5	13.6
C18:3n-3	64.5	264.0	295.2	307.9	63.1	269.8	299.5	304.5	32.1
C20:4n-6	8.1	6.6	6.4	7.4	8.6	4.9	6.6	7.3	0.7
C20:5n3	9.2	7.5	7.2	8.3	7.4	5.3	7.7	8.4	0.6
C22:5n3	4.5	4.5	5.3	4.7	4.3	6.5	5.0	4.4	0.6
C22:6n3	7.4	10.1	9.6	10.9	6.8	9.6	8.7	10.7	0.7
PUFAs^3)^	476.7	491.3	524.9	535.1	469.5	490.2	528.7	539.0	38.3
n-3^4)^	85.6	286.1	317.3	331.8	81.6	291.2	320.9	328.4	19.5
n-6^5)^	384.8	212.1	210.4	209.2	397.8	215.3	215.9	207.8	21.5
n-6/n-3	4.49	0.74	0.61	0.62	4.87	0.74	0.61	0.61	0.05

SDM, the standard deviation of the means.

1) SFAs, saturated fatty acids: sum of all fatty acids without double bonds; includes C17 and C20, in addition to individually reported SFAs.

2) MUFAs, monounsaturated fatty acids: sum of all fatty acids with a single double bond; includes C14:1n5, C18:1n7 and C22:1n9 in addition to individually reported MUFAs.

3) PUFAs, polyunsaturated fatty acids: sum of all fatty acids with ≥2 double bonds; includes C20:3n3 and individually reported PUFAs.

4) n-3 fatty acids: sum of all fatty acids with 3 carbon atoms double bonds and individually reported PUFAs; include C20:3n-3, C20:5n3, C22:6n3, C18:3n-3.

5) n-6 fatty acids: sum of all fatty acids with 6 carbon atoms double bonds and individually reported PUFAs; include C20:4n-6, C18:2n-6.

**Table 3:** The results of two-way analysis of variance analysis for measured flaxseed-herbs effect

Items	p-value			Main effects (pooled means)					
	
	Herbs effect	Flaxseed effect	Interactions	Without herbs	With herbs	Control	FLX9s	FLX18s	FLX27s
Egg production	0.049	0.155	0.250	73.97	77.50				
C:14	0.819	0.050	0.988			9.50^B^	10.40^AB^	10.10^AB^	11.75^A^
C18:0	0.785	0.050	0.794			93.96^B^	104.25^A^	95.82^AB^	91.98^B^
SFA	0.798	0.051	0.969			383.7^A^	359.9^AB^	350.5^B^	343.8^B^
C16:1n-9	0.813	0.000	0.752			24.05^A^	14.65^B^	12.85^B^	12.60^B^
C18:1n-9	0.855	0.001	0.996			388.2^A^	351.2^B^	340.3^BC^	319.6^C^
MUFA	0.847	0.002	0.998			414.5^A^	381.7^B^	357.2^BC^	348.9^C^
C18:2n-6	0.997	0.000	0.985			135.3^A^	101.9^B^	92.9^B^	93.8^B^
C18:3n-3	0.964	0.000	0.838			16.6^D^	84.2^C^	110.3^B^	133.2^A^
C20:4n-6	0.441	0.000	0.912			11.30^A^	7.65^B^	7.25^B^	7.70^B^
C20:5n-3	0.511	0.000	0.923			4.28^B^	6.50^A^	7.20^A^	6.60^A^
C22:6n-3	0.994	0.001	0.979			5.15^B^	10.22^A^	11.45^A^	12.20^A^
PUFA	0.790	0.000	0.963			171.9^C^	214.3^B^	235.8^AB^	258.6^A^
n-3	0.948	0.000	0.944			26.0^D^	100.8^C^	128.8^B^	152.0^A^
n-6	0.928	0.000	0.983			146.6^A^	109.8^B^	100.1^B^	101.6^B^
n-6/n-3	0.622	0.000	0.806			5.64^A^	1.08^B^	0.77^C^	0.66^C^
Yolk cholesterol	0.002	0.050	0.613	12.37	10.62	12.62^A^	11.71^AB^	11.01^B^	10.63^B^
Total cholesterol	0.001	0.087	0.753	239.1	210.4				
Yolk HDL	0.000	0.696	0.180	3.36	4.40				
Glucose	0.050	0.285	0.934	283.7	258.7				
Blood LDL	0.000	0.000	0.249	79.5	66.2	81.0^A^	69.1^C^	75.9^B^	65.4^C^
SOD	0.000	0.593	0.560	71.46	93.67				
GPx	0.000	0.001	0.227	885	1,021	1,002^A^	988^A^	983^A^	840^B^
WBC	0.011	0.713	0.981	17.60	19.55				
MCH	0.315	0.000	0.060			30.21^C^	40.11^A^	34.09^B^	29.05^C^

SFA, saturated fatty acids; MUFA, monounsaturated fatty acids; PUFA, polyunsaturated fatty acids; HDL, high-density lipoprotein; LDL, low-density lipoprotein; SOD, superoxide dismutase; GPx, glutathione peroxidase; WBC, white blood cell; MCH, mean corpuscular haemoglo-bin.

When the p-value of interaction was not significant, we compared the pooled main effects: Flaxseed Effect via Tukey test and Herbs Effect via the Independent Samples T-Test (p<0.05). When the p-value of interaction was significant, we unpacked the effects via Tukey and the Inde-pendent Samples T-Test, respectively which was shown with subsets in Tables (Table 6–8). The non-significant parameters were not reported.

A–D The letters indicate significant differences among groups.

**Table 4:** Egg performance of laying hen fed test diets with different levels of extruded flaxseed with and without herbal medicine for nine weeks

Items	Control	Flx9	Flx18	Flx27	Control+H	Flx9+H	Flx18+H	Flx27+H	SDM
Shell thickness (mm)	0.29	0.29	0.26	0.25	0.26	0.30	0.26	0.27	0.06
Shell strength (kg/cm^2^)	3.32	3.28	3.33	3.29	3.24	3.29	3.30	3.31	0.45
Egg weight (g)	65.74	65.88	65.92	65.06	65.39	66.42	66.30	66.01	4.36
Haugh unit^1)^	79.64	75.31	79.99	82.12	80.64	81.03	76.55	80.33	3.64
Yolk weight (g)	20.38	19.45	19.52	19.67	19.65	20.00	20.23	19.85	2.43
Egg production^2)^ (%)	75.05	75.02	74.14	71.74	72.49	81.44	80.91	75.17	4.23
Feed intake (g/d/hen)	108.91	104.21	103.82	103.77	106.55	108.49	107.12	109.70	5.92
Egg mass^3)^ (g/d)	49.33	49.43	48.87	46.67	47.40	54.09	53.64	49.61	3.65
Feed conversion ratio^4)^	2.21	2.17	2.12	2.22	2.25	2.00	1.99	2.21	0.39

Values are represented by means of twelve samples; Interaction Effect (herbs-flaxseed) was not significant for the data of this table.

SDM, the standard deviation of the means.

1) Haugh unit: HU = 100×log 10 (h–1.7w^0.37^+7.6); h, albumin height (mm); w, egg weight (g).

2) Egg production = (total produced egg/days)×100.

3) Egg mass = (egg production×egg weight)/100.

4) Feed conversion ratio = feed intake/egg mass.

**Table 5:** Yolk fatty acid profiles of laying hens were fed test diets with different levels of extruded flaxseed with and without herbal medicines for nine weeks

Items	g/100 g fatty acid methyl esters (FAMEs)								SDM

	Control	Flx9	Flx18	Flx27	Control+H	Flx9+H	Flx18+H	Flx27+H	
C14:0	9.4	10.5	10.0	11.6	8.6	10.3	10.2	11.9	1.6
C16:0	264.6	243.7	237.5	236.1	260.4	247.2	234.9	230.5	16.5
C18:0	96.5	103.5	96.5	90.9	90.8	105.0	95.1	93.1	7.4
∑SFA^1)^	388.0	361.0	352.8	341.4	379.5	358.9	348.3	346.3	28.7
C16:1n-9	24.5	13.7	12.8	12.7	23.6	15.6	12.9	12.5	3.4
C18:1n-9	386.3	350.1	339.1	320.6	390.1	352.4	341.6	318.7	20.1
∑MUFA^2)^	413.1	379.1	357.2	348.9	416.3	384.3	357.2	349.0	25.4
C18:2n-6	134.5	101.2	92.9	95.4	136.2	103.1	92.7	92.3	16.7
C18:3n-3	17.2	84.5	107.3	134.8	15.9	83.9	113.0	131.6	12.3
C20:4n-6	10.9	7.7	7.1	7.5	11.7	7.6	7.4	7.9	1.1
C20:5n-3	4.1	6.3	7.0	6.7	4.5	6.7	7.4	6.5	1.6
C22:6n3	5.0	10.6	11.4	12.0	5.3	9.5	11.5	12.4	2.8
∑PUFA^3)^	171.4	214.8	235.6	263.3	172.5	213.9	236.1	254.0	21.4
∑n-3^4)^	26.3	101.4	125.7	153.5	25.7	100.3	131.9	150.5	18.1
∑n-6^5)^	145.4	108.9	100.0	102.9	147.9	110.7	100.1	100.2	12.5
n-6/n-3	5.53	1.07	0.79	0.67	5.75	1.10	0.76	0.66	0.1

Values are represented by means of four samples; the Interaction Effect (herbs-flaxseed) was not significant for the data of this table.

SDM, the standard deviation of the means.

1) SFAs, Saturated fatty acids: the sum of all fatty acids without double bonds, includes C17:0 and C20:0, in addition to individually reported SFAs.

2) MUFAs, monounsaturated fatty acids: the sum of all fatty acids with a single, double bond; includes C14:1n5, C18:1n7, and C22:1n9 in addition to individually reported MUFAs.

3) PUFAs, polyunsaturated fatty acids: the sum of all fatty acids with ≥2 double bonds; includes C20:3n3 and individually reported PUFAs.

4) n-3 fatty acids: sum of all fatty acids with 3 carbon atoms double bonds and individually reported PUFAs; include C20:3n-3, C20:5n3, C22:6n3, C18:3n-3.

5) n-6 fatty acids: sum of all fatty acids with 6 carbon atoms double bonds and individually reported PUFAs; include C20:4n-6, C18:2n-6.

**Table 6:** Egg lipid components and blood biochemistry of laying hen were fed test diets with different levels of flaxseed with and without herbal medicines for nine weeks

Items	Control	Flx9	Flx18	Flx27	Control+H	Flx9+H	Flx18+H	Flx27+H	SDM
Yolk CHO (mg/g yolk)	13.01	12.95	12.12	11.39	12.24	10.47	9.90	9.87	1.52
Yolk TG (mg/g yolk)	194.2	200.5	187.9	190.1	195.3	179.4	201.8	170.0	18.1
Total CHO (mg)	249.1	238.7	239.9	228.6	232.4	201.5	207.1	200.8	17.9
Total TG (mg)	3,354.3	3,379.7	3,340.0	3,568.8	3,326.9	3,346.6	3,470.1	3,322.5	158.6
Yolk HDL (mg/g yolk)	3.67	3.23	3.18	3.36	3.93	4.89	4.27	4.51	0.61
Yolk LDL (mg/g yolk)	8.03	8.00	7.92	7.64	8.15	7.77	6.99	7.10	0.92
Total protein (mg/dL)	5.12	5.34	4.92	5.00	4.58	5.25	4.93	4.55	0.75
Albumin (mg/dL)	1.39	0.97	1.20	1.31	1.46	1.24	0.99	1.11	0.32
Globulin (mg/dL)	2.97	3.07	3.05	2.74	3.02	3.15	3.26	3.01	0.81
A/G ratio	0.47	0.32	0.39	0.47	0.48	0.39	0.30	0.37	0.1
Glucose	297.8	291.3	275.5	270.3	282.3	255.8	246.6	250.1	29.4
Blood CHO (mg/dL)	205.3^a*^	194.5^b*^	180.5^b^	179.3^b^	175.8	166.4	179.3	157.0	8.9
Blood TG (mg/dL)	1,345.4	1,311.7	1,287.9	1,265.4	1,300.1	1,294.6	1,369.5	1,277.2	160.0
Blood HDL (mg/dL)	52.2^b*^	61.6^ab*^	52.7^b*^	65.4^a*^	76.3	70.5	71.8	76.6	4.9
Blood LDL (mg/dL)	86.5	78.1	80.3	73.2	75.5	60.1	71.5	57.7	5.5

Values are represented by means of four samples.

SDM, the standard deviation of the means; CHO, cholesterol; TG, triglyceride; CHO, cholesterol; HDL, high-density lipoprotein; LDL, low-density lipoprotein. blood CHO; blood cholesterol in serum; blood TG, blood triglyceride in serum; blood HDL, blood high density lipoprotein; blood LDL, blood low density lipoprotein.

a,b The letters indicate significant differences in the four treatments without herbs when the interaction (herbs-flaxseed) was significant (p<0.05).

* The asterisk shows significant different between with herbs and without herbs for each flaxseed level (p<0.05).

**Table 7:** Antioxidant and serological enzymes of laying hen were fed test diets with different levels of ex-truded flaxseed with and without herbal medicines for nine weeks

Items	Control	Flx9	Flx18	Flx27	Control+H	Flx9+H	Flx18+H	Flx27+H	SDM
MDA (nmol/mL)	10.4	9.3	11.3	13.4	13.9^x^	7.4^y^	7.1^y^^*^	7.0^y^^*^	1.0
SOD (U/mL)	74.6	65.4	76.8	69.1	92.0	90.5	92.8	99.2	8.2
GPx (U/mL)	969.4	880.5	907.7	782.0	1,034.3	1,095.6	1,057.1	896.8	48.9
GRx (U/mL)	826.5^a^	894.3^a^	853.6^a^	645.5^b*^	810.7	822.5	800.2	797.7	62.0
CAT (U/mL)	8.2	7.8	8.0	7.9	8.2	9.0	7.7	8.0	0.9
LDH (U/L)	642.0	598.4	624.5	600.3	629.8	600.0	634.6	610.3	40.4
ALP (U/L)	115.7	110.0	133.5	120.8	105.3	132.3	122.7	127.4	9.7
AST (U/L)	89.6^b^	135.1^a*^	100.2^a*^	142.2^a*^	93.5	80.7	84.5	80.5	6.4
ALT (U/L)	35.6^b^	63.2^a*^	70.4^a*^	67.7^a*^	31.6	31.4	39.3	32.1	4.3

Values are represented by means of four samples.

SDM, the standard deviation of the means; MDA, malondialdehyde; SOD, superoxide dismutase; GPx, glutathione peroxidase; GRx, gluta-thione reductase; CAT, catalase; LDH, lactate dehydrogenase; ALP, alkaline phosphatase; AST, aspartate transaminase; ALT, alanine trans-aminase.

a,b The letters indicate significant differences in without herbs.

x,y The letters indicate significant differences in with herbs groups when the interaction (herbs-flaxseed) was significant (p<0.05).

* The asterisk shows a significant difference between with herbs and without herbs for each flaxseed level (p<0.05).

**Table 8:** Haematology and immune response of laying hen were fed test diets with different levels of extruded flaxseed with and without herbal medicines for nine weeks

Items	Control	Flx9	Flx18	Flx27	Control+H	Flx9+H	Flx18+H	Flx27+H	SDM
Total antibody 24 days	4.68^a^	4.00^a*^	3.85^a*^	2.01^b*^	4.89	5.24	5.11	4.12	0.45
IgG 24 days	2.32^a^	2.61^a*^	1.28^b*^	0.98^b*^	2.36	1.99	2.16	2.13	0.24
IgM 24 days	2.16^a^	1.39^b*^	2.57^a^	1.03^b*^	2.53^yz^	3.25^x^	2.95^xy^	1.99^z^	0.25
Total antibody 42 days	6.20^a^	4.93^ab*^	4.00^ab*^	3.19^b*^	5.12	5.87	6.56	5.50	0.82
IgG 42 days	2.96^a*^	3.14^a^	1.95^b*^	1.30^c*^	2.54^yz^	3.01^x^	3.19^x^	1.98^z^	0.15
IgM 42 days	3.24^a^	1.99^b*^	2.05^b*^	1.89^b*^	2.58^y^	2.85^y^	4.37^x^	3.52^xy^	0.41
Hematocrit (%)	29.30	27.19	29.94	27.05	28.63	29.12	29.00	28.55	2.00
Hemoglobin (g/dL)	6.74^a^	7.12^a^	6.45^ab^	5.40^b^	5.96	6.97	7.15	6.27	0.53
RBC (10^6^/mm^3^)	2.15	1.75	1.88	2.15	2.01	1.76	2.11	1.90	0.19
WBC (10^3^/mm^3^)	17.5	18.0	17.7	17.1	19.3	19.8	20.2	18.9	1.6
MCV (fL)	133.79	155.37	159.25	125.81	142.43	165.45	137.44	150.26	10.9
MCH (pg)	30.77	40.68	34.31	25.11	29.65	39.54	33.89	33.00	4.2
MCHC (%)	23.00	25.26	21.54	19.96	20.82	23.83	24.65	21.96	3.4
Blood performance	15.15	14.95	14.93	14.76	14.91	15.09	15.28	14.87	0.53

Values are represented by means of four samples.

SDM, the standard deviation of the means; IgG, immunoglobulin G; IgG, immunoglobulin M; RBC, red blood cell; WBC, white blood cell; MCV, mean corpuscular volume; MCH, mean corpuscular haemoglobin; MCHC, mean corpuscular haemoglobin concentration.

a–c The letters indicate significant differences in without herbs (p<0.05).

x–z The letters indicate significant differences in with herbs groups when the interaction (herbs-flaxseed) was significant (p<0.05).

* The asterisk shows a significant difference between with herbs and without herbs for each flaxseed level (p<0.05).
